# Developing a well-received pre-matriculation program: the evolution of MedFIT

**DOI:** 10.1007/s44217-022-00012-z

**Published:** 2022-09-15

**Authors:** Alexa Allen, Brandon Conner, Brooke Gantman, Kendal Warner, Ranna Nash, Brady Janes, Megan Hellum, Cherie Singer

**Affiliations:** 1 Medical Student, University of Nevada, Reno School of Medicine, Reno, NV USA; 2 Coordinatior for Student Development and Academic Enrichment, University of Nevada, Reno School of Medicine, Reno, NV USA; 3 Director of Student Affairs, University of Nevada, Reno School of Medicine, Reno, NV USA; 4 Director of Curricular Design, University of Nevada, Reno School of Medicine, Reno, NV USA; 5 Associate Dean, Office of Admission and Student Affairs, University of Nevada, Reno School of Medicine, Reno, NV USA

## Abstract

**Background:**

The transition to medical school marks a very stressful time for matriculating students. Despite this challenging transition period, intellectually rigorous pre-matriculation programs are not a common component of the curriculum at many medical schools across the country. Students are often not given the opportunity to learn about the high expectations of medical school before being thrown into classes. Thus, more time and attention should be devoted to curricular interventions that target this critical window in medical education.

**Methods:**

MedFIT is a robust, 1.5-week orientation program that introduces matriculating students to the rigors of medical school in a low-stakes environment. This program provides students with a preview of the University of Nevada, Reno School of Medicine (UNR Med) curriculum through hands-on learning sessions in addition to exposing them to extracurricular opportunities and research initiatives. Furthermore, MedFIT connects incoming students with second-year mentors, laying the foundation for longitudinal peer mentorship.

**Findings:**

Qualitative survey feedback from each subsequent class has been increasingly positive, demonstrating MedFIT’s effectiveness in improving students’ academic and interprofessional transitions into medical school. Despite the program's hybrid format in 2020 due to COVID-19, overall ratings were as positive as the previous year. Additionally, students had decreased rates of remediation, repeated years, and withdrawal, and had better Match outcomes when compared to students who participated in UNR Med’s previous orientation program.

**Discussion and conclusions:**

MedFIT remains a well-received, adaptable entity that is continuously modified every year to best supplement student needs and mirror the pre-clinical curricular landscape present at that time.

**Supplementary Information:**

The online version contains supplementary material available at 10.1007/s44217-022-00012-z.

## Background

The transition to medical school is an incredibly challenging and stressful time for incoming students [1]. Interventions and programs aimed at improving the transition periods that medical students face throughout their education are not a novel idea [2]. However, there is a focus on the transition from pre-clinical to clinical training years and from medical school to residency, with only a recent emphasis being placed on the pre-matriculation period and the transition to medical school [3]. Pre-matriculation programs and courses have become increasingly popular in medical schools across the United States and aim to take a diverse group of new medical students and prepare them for the next four years [4]. Many of these programs focus on targeting students who are deemed to be at-risk and/or underrepresented in medicine, which is defined by the Association of American Medical Colleges (AAMC) as ethnic populations that are underrepresented in the medical profession relative to their numbers in the general population [5, 6]. While there is significant variation in the structure, length, and content included in these programs, efforts like these that are geared toward improving the transition for incoming first-year medical students have been shown to lead to better integration, progression, and retention, despite the stress and rigor associated with pre-clinical medical education [1, 4, 7].

The Medical Freshman Intensive Transition Program, better known as MedFIT, is a 1.5-week, skills-based program at the University of Nevada, Reno School of Medicine (UNR Med) that provides incoming first-year students with exposure to the rigorous curriculum, professional responsibilities, and new learning environment of a medical student before officially beginning their training. The evolution of this pre-matriculation program began with efforts to combine UNR Med’s Medical Student Bridge Program (MSBP) with the former Orientation Week. The MSBP was initially designed to target individuals deemed “at-risk” for potentially having to remediate a curricular block or repeat an entire pre-clinical year. In contrast, all students participated in Orientation Week, which served to cover housekeeping topics such as advising, remediation, sexual harassment training, etc. However, in the fall of 2016, first-year students in the MSBP proposed that the whole incoming class participate in the program as they found it to be extremely beneficial to their transition into medical school. Given the improvement in remediation and retention rates that were seen with students who participated in the MSBP as well as their positive feedback, the MedFIT program was created. This program aims to integrate professional identity development, team building activities, and an introduction to the curriculum in order to create a low-stakes but high-intensity environment for students.

When MedFIT was introduced in 2017, the program was planned and facilitated by faculty members. Throughout its evolution, however, there has been an increasing interest in medical student involvement in both the planning and delivery of the program. In 2018, a cohort of medical students was recruited during their first year to serve as “pack mentors” who played an integral role in the planning and execution of MedFIT for the next class of first-year students. In addition to their role facilitating the program's various activities and curricular components, pack mentors also serve as peer mentors. The implementation of this formal peer mentorship program was largely influenced by student responses to satisfaction surveys that demonstrated a need for additional guidance for incoming medical students. Peer mentorship has been shown to play an essential role in both the personal and professional development of medical students during their education and is an effective way of increasing both social and academic support for new students during their transition to medical school and beyond [1, 8–10].

This paper aims to present and describe the MedFIT pre-matriculation program offered to all first-year medical students at UNR Med. Emphasis will be placed on the curricular structure of the program in conjunction with the peer mentorship component. Additionally, the changes that were made to this program due to COVID-19 will be discussed to showcase its adaptability in having to adhere to new social distancing and virtual learning guidelines in the face of a global pandemic.

## Methods

### Development of MedFIT

MedFIT has served as the 1.5-week, mandatory orientation program for matriculating MD students at UNR Med since 2017 and is supported by the AAMC. The program is formally termed MED 630 on the academic transcript, and grading is based on a Pass/Fail scheme. Although this paper focuses on MedFIT for the 2019–2020 and 2020–2021 academic years, this program will continue as UNR Med’s pre-matriculation program for the foreseeable future.

The Global MedFIT Committee oversaw the planning and implementation process of this program, whose members consisted of the Associate Dean of Admissions and Student Affairs, Director of Student Affairs, Coordinator for Student Development and Academic Enrichment, Associate Dean of Diversity & Inclusion, curriculum directors/associate professors, and MS1-MS4 students who served in the role of “head pack mentor” during MedFIT 2018, 2019, and 2020. This committee was responsible for creating the schedule, orchestrating the program's curricular layout, integrating student feedback on an annual basis, and serving as the liaison between students and administration.

Each year, a group of approximately 20–23 first-year medical students was recruited via a formal application process to serve as pack mentors for the next incoming class of students. A mentorship agreement was signed by each pack mentor before their onboarding experience conducted by the Office of Admissions and Student Affairs. All pack mentors received a one-time stipend of approximately $500 that was provided as part of the MedFIT orientation budget through the office of Admissions and Student Affairs.

Pack mentors were divided into several sub-committees and worked with the head mentors to create, modify, and develop various components of the orientation program. These five sub-committees included: (1) Content, (2) learning strategies, (3) collaboration, professionalism, and advising, (4) extracurriculars, and (5) nutrition and wellness. Three to four pack mentors served on each sub-committee and met at various points throughout the spring and summer prior to that year’s MedFIT program in August.

**Fig. 1:**
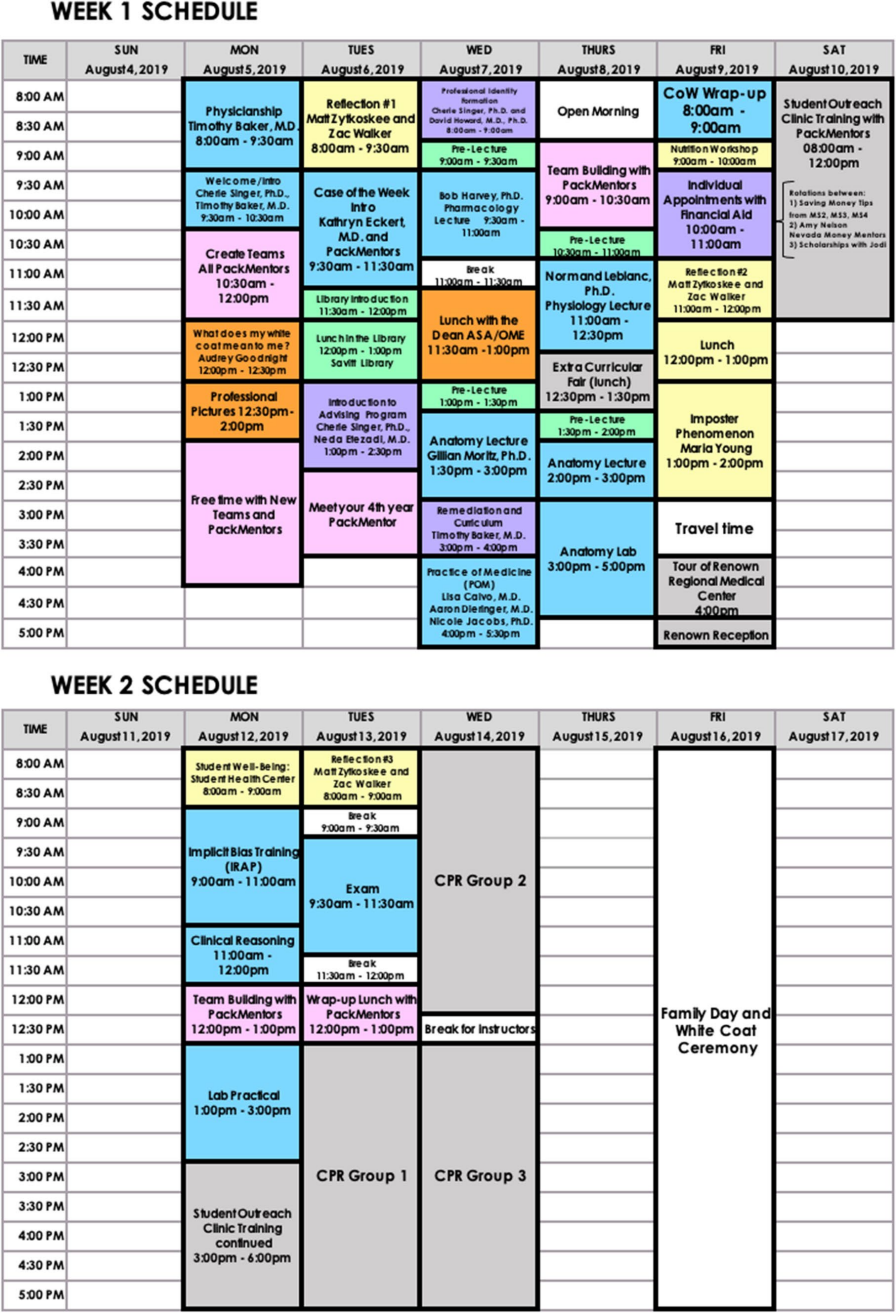
Overview of the MedFIT 2019 orientation program schedule for the class of 2023. Blue boxes represent activities planned by the Content Sub-Committee, green boxes planned by the Learning Strategies Sub-Committee, gray boxes planned by the Extracurriculars Sub-Committee, yellow boxes planned by the Nutrition & Wellness Sub-Committee, purple boxes planned by the Collaboration, Professionalism, & Advising Sub-Committee, and orange boxes planned by the Global MedFIT Sub-Committee. Pink boxes represent dedicated peer mentorship time

The Content Sub-Committee worked alongside associate professors to prepare gross anatomy, histology, and physiology lectures. They created a “mock” anatomy lab practical and a “mock” written exam that covered the topics discussed in the lectures during the orientation program. They also coordinated workshops that focused on clinical case vignettes, basic H&P skills, clinical note writing skills, and implicit bias training. The Learning Strategies Sub-Committee created PowerPoint presentations, handouts, and informational sessions that revolved around study resources often utilized in the pre-clinical years such as Anki, AMBOSS, Boards and Beyond, Sketchy Medical, etc. The Collaboration, Professionalism, and Advising Sub-Committee designed various team-building activities meant to enhance class collaboration and camaraderie. They also organized professionalism, remediation, and advising workshops hosted by the Associate Dean of Admissions and Student Affairs and the Associate Dean of Medical Education. The Extracurriculars Sub-Committee orchestrated an extracurriculars conference to promote student interest groups, committees, research opportunities, and other positions available to students outside of the clinical learning environment. These students also assisted faculty with scheduling financial aid sessions, a library resource workshop, CPR training sessions, Student Outreach Clinic (SOC) training, and a Renown Regional Medical Center tour. The Nutrition and Wellness Sub-Committee coordinated the purchase, set-up, tear-down, and organization of the daily breakfast bar and various lunch sessions provided to the matriculating students and pack mentors. They also collaborated with faculty in order to introduce UNR Med’s integrated pre-clinical nutrition curriculum. A sample schedule from MedFIT 2019 is shown below in Fig. 1 and demonstrates that students participated in curricular activities in addition to general orientation sessions.

### Development of the peer mentorship component

Pack mentors not only assisted with content development for MedFIT but were also tasked with serving in a mentorship capacity for 3–4 incoming first-year medical students to foster personal and professional growth. A total of three trainings were held for PackMentors before the start of MedFIT. This included an “onboarding” session in December, a team building-focused training in May, and a final training and debrief session several days before MedFIT officially began. One of the primary goals of these training sessions was to prepare these students to serve as effective pee mentors to the incoming first year students. During these trainings, we introduced and reviews important communication skills and strategies that will aid in building valuable mentorship relationships through presentations, facilitated group discussions, and small group exercises.

After the start of MedFIT, pack mentors met with their mentees multiple times during the program to answer questions, share personal experiences related to studying and time management, and discuss other aspects related to success during the first year of medical school. Beyond this, mentors were expected to continue this role longitudinally by reaching out to their mentees before exams and at the end of each curricular block, during second year for STEP 1 preparation, and during third year when students begin pondering residency and specialty selection.

### Data collection and program feedback

Longitudinal academic outcomes data was collected and analyzed for the Class of 2021, 2022, and 2023. The academic outcomes assessed included remediation, repeated years, withdrawal, and Match results at the end of the fourth year. Remediation was defined as students who failed to meet the requirements established for the course and therefore, were required to repeat that specific course under administrative supervision. The repeated years category included any student who was permitted to repeat a full academic year. Withdrawal represented any student who formally withdrew from the program. Finally, outcomes in the Match were evaluated and the number of students who did not successfully match was recorded. It is important to note that the Match outcomes data only includes students who successfully made it to graduation for a given class; therefore, it does not include students who delayed graduation for any reason during their program of study. Similarly, longitudinal outcomes data was unavailable for the Class of 2024 and was incomplete for the Class of 2022 and 2023 because this data is not yet available.

At the conclusion of MedFIT each fall, quantitative and qualitative data about the overall program as well as the mentorship component is collected via a comprehensive survey. Students also complete follow-up surveys after their fall and spring semesters to evaluate the program’s impact on student success throughout the first year of medical school. A thematic analysis of the qualitative narrative data that is collected in these surveys was conducted with a focus on the following main topics: curricular structure, academic rigor, expectations, and extracurricular opportunities. A summary of results from this analysis have been compiled and are shown below in Table 2. All materials were submitted to the Institutional Review Board (IRB #1516448-1) at the University of Nevada, Reno and were determined to be exempt.

### COVID-19 changes

In response to the COVID-19 pandemic, the program was converted to a hybrid format. Most of the sessions were delivered virtually and any in-person clinical skills sessions were conducted as similarly as possible to previous years but with appropriate social distancing measures in place. The incoming medical students were still able to meet with their pack mentors virtually, in addition to a few in-person opportunities.

## Findings

### Longitudinal academic outcomes

Longitudinal academic outcomes data, including the number of students who remediated, repeated an academic year, or withdrew, was recorded for all students who participated in the MSBP and MedFIT orientation programs as shown below in Table 1. Data showed that an average of 22.7% of students from the Class of 2018 (n = 17), 2019 (n = 23), and 2020 (n = 20) who were deemed to be “at risk” and subsequently participated in the MSBP program completed some form of remediation. In contrast, a lower rate of remediation was seen after the implementation of MedFIT with an average of 15.0% from the Class of 2021 (n = 73), 2022 (n = 71), and 2023 (n = 70). Similarly, the average number of students who repeated an academic year was 18.3% for the three cohorts who participated in MSBP and 5.1% for the first three classes of students who participated in MedFIT as this data has not yet been finalized for the Class of 2024. Of the students who participated in the MSBP program, an average of 8.3% withdrew and an average of 8.5% did not match. Because the Class of 2021 and 2022 are the only groups of students who have both participated in MedFIT and graduated, these are the only classes for which withdrawal and match outcomes have been finalized. Of these students, an average of 3.5% withdrew and 1.8% did not match (Table 1).

**Table 1:** Longitudinal academic outcomes data including remediation, repeated years, withdrawal, and match results for students who participated in the Medical Student Bridge Program (MSBP) vs. the MedFIT Orientation Program

	MSBP^a^				MedFIT^b^			
Outcome assessed	Class of 2018 (n = 17) (%)	Class of 2019 (n = 23) (%)	Class of 2020 (n = 20) (%)	Average (%)	Class of 2021 (n = 73) (%)	Class of 2022 (n = 71) (%)	Class of 2023 (n = 70) (%)	Average (%)
Remediation^c^	17.6	30.4	20.0	22.7	40.0	11.3	17.1	15.0
Repeat^d^	0.0	21.7	30.0	18.3	5.5	4.2	5.7	3.5
Withdrawal^e^	23.5	4.3	0.0	8.3	5.5	1.4		15.0
Did not match^f^	7.7	6.7	10.5	8.5	3.6	0.0		1.8

^a^MSBP (Medical Student Bridge Program)

^b^MedFIT (Medical Freshman Intensive Transition Program)

^c^Remediation: Includes any student who failed to meet the requirements established for the course and therefore, were required to repeat that specific course under administrative supervision

^d^Repeat: Includes any student who was permitted to repeat a full academic year during their program of study

^e^Withdrawal: Includes any student who formally withdrew from the program at any point

^f^Did not match: Includes all students who did not successfully match. It is important to note that the match outcomes data only includes students who successfully reached graduation for a given class, and therefore, does not include students who withdrew at any point during their program of study for any given reason

### Student satisfaction and responses

**Fig. 2:**
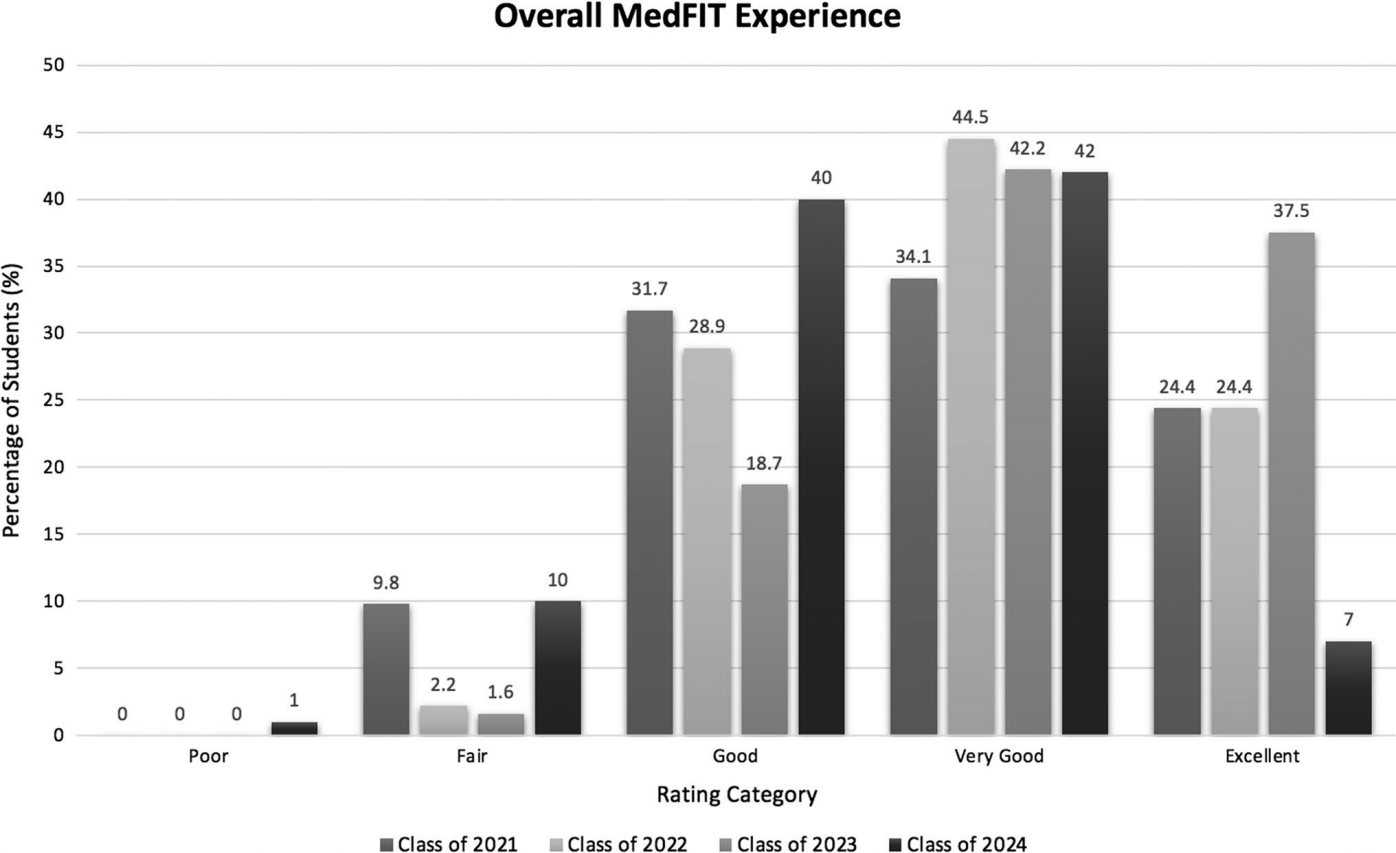
Percentage of first-year medical students from the class of 2021, 2022, 2023, and 2024 who rated their overall MedFIT experience as “poor”, “fair”, “good”, “very good”, or “excellent”

MedFIT is a consistently well-received program year-to-year, with the majority of students rating their overall experience as “good” or above, as shown below in Fig. 2. Despite the hybrid format of MedFIT 2020 in the face of COVID-19, the same percentage of students rated their overall experience as “good” or above when compared to the previous year; however, their responses were skewed more towards “good” and “very good” instead of “excellent,” as was seen in previous years.

Primarily, students valued how lecture sessions, anatomy labs, clinical vignette sessions, exams, and clinical skills courses were introduced during the low-stakes environment of MedFIT before the semester and grading officially began. Additionally, students felt that the sessions were well-organized, and fall semester follow-up surveys indicated that the sessions provided insight into a typical day during the year. In terms of academic and professional expectations throughout medical school, students appreciated learning about the Medical Student Performance Evaluation (MSPE), remediation policies, professional identity development, and the numerous opportunities available for involvement outside of the classroom via an extracurriculars conference. Furthermore, students commended the approachability and accessibility of the associate deans, advisors, and learning specialists, and regarded the SOC—UNR Med’s free clinic to the public—training as the highlight of MedFIT.

The peer mentorship component of MedFIT has increased student satisfaction with the program at large and their overall first-year experience at the medical school. In general, student responses from the Class of 2023 and 2024 showed that the pack mentor program provided a sense of community, as opposed to the more “cutthroat” medical school environment that they had initially expected. Having peer mentors intermixed throughout the orientation alleviated some of the anticipated stress, created a supportive environment, and provided a wealth of resources for first-year students as they made this transition. Pack mentors helped keep students in the Class of 2024 informed about the most updated COVID-19 guidelines and consistently followed appropriate distancing measures, setting a strong example of the ethical responsibility that medical students have as future healthcare professionals in the face of a global pandemic. Overall, the presence of pack mentors helped reassure incoming students that they were receiving a similarly conducted MedFIT as was done in previous years, even though there was a virus controlling what was and was not possible. Specific quotes from students in the Class of 2023 and 2024 regarding key components of the MedFIT program can be found in Table 2.

**Table 2:** Select quotes from first-year medical students in the Class of 2023 and 2024 regarding key components of the MedFIT orientation program

Survey responses from first-year medical students	
Curricular structure	“I enjoyed COW^a^, POM^b^, and anatomy lab the most. The hands-on movement and team participation allowed me to start thinking critically and apply the vast resources that were discussed in the content/pre-lecture sessions.”—Class of 2023 “… it finally felt like we were really in medical school, getting to do the things we've been waiting to do our whole lives.”—Class of 2024 “These seemed to be the most helpful sessions because they felt like a real glimpse into what medical school would be like. They were the most relatable.”—Class of 2024
Academic rigor	“It was nice to see what test questions would look like and it gave me a better idea of how to prepare for future exams.”—Class of 2023 “I liked that I didn't have to worry too much about the grade and could focus on familiarizing myself with the format of the [NBME platform].”—Class of 2023
Expectations	“These sessions answered many questions I had about expectations and support throughout the school year.”—Class of 2023 “These workshops in particular offered a lot of information that I was wondering about before orientation. I liked how I was made aware of the resources that are available to me and how to access them early on.”—Class of 2024
Extracurricular opportunities	“I loved hearing more about the student health center, library resources, financial aid, etc. I really enjoyed the extracurricular fair because we got to speak with a lot of MS2s about different SIGs^c^ and how we can get more involved during the next few years.”—Class of 2023 “[The] extracurricular fair was [an] awesome way to hear about the different opportunities available to us. I enjoyed having the people who served in various positions speak to us. That was really helpful.”—Class of 2024
Peer mentorship program	“I love this program and think it’s an amazing way to help new medical students transition to the atmosphere of medical school as well as building their social circles with students from other classes, not just students from the same class.”—Class of 2023 “All of the pack mentors were excited to be there, and it showed. They welcomed questions and were always looking for ways to help us MS1s out.”—Class of 2023 “All pack mentors were very welcoming. I felt like I was able to talk to any of them even if they weren't my designated pack mentor.”—Class of 2024
Noteworthy qualities of pack mentors	“No sense of indoctrination or a ‘sink or swim’ mentality”—Class of 2024 “Willingness to share the positive and negative aspects of med school. Very honest.”—Class 2023 “Appreciated the realistic suggestions such as avoiding burnout and not spreading oneself too thin”—Class of 2024 “She checked in with me after each exam and offered to sit down and talk about how I was feeling. It was nice knowing that someone was there after that weird period post-exam where you don't know how others did.”—Class of 2023

^a^COW (Case of the Week): UNR Med’s version of case vignette workshops for first-and second-year students

^b^POM (Practice of Medicine): UNR Med’s clinical skills course for first-year students

^c^SIGs (Student Interest Groups)

## Discussion

### Effectiveness, evolution, and impact of MedFIT

Since the implementation of MedFIT in 2017, data has shown overall improvement in longitudinal academic outcomes when compared to the former MSBP program. Average rates of remediation, repeated years, and withdrawal were lower for the students who participated in the required MedFIT program, compared to those who went through the MSBP program offered only to students deemed “at-risk”. Additionally, match outcomes for the Class of 2021 and 2022 showed increased success compared to the three classes with students who participated in the MSBP program. These findings indicate that the implementation of MedFIT has improved student success in the Match overall and has significantly impacted student retention throughout the four years of medical school.

As demonstrated by student feedback each year, MedFIT continues to be an informative and engaging introduction to medical school that allows all matriculating students to participate as opposed to other pre-matriculation programs that focus on students deemed “at-risk” of struggling academically or students from historically disadvantaged groups [5, 6]. In general, annual student feedback helps determine whether sessions should be added, removed, or changed in their entirety. One example of this involves the Medical Student Performance Evaluation (MSPE) introduction. The Class of 2022 was not introduced to the MSPE until late in the fall semester of their first year. Most individuals in the class indicated that they would have preferred learning about the MSPE during MedFIT, which may have encouraged them to become more involved in extracurricular/professional opportunities early on. Thus, an MSPE session was added to the MedFIT program for the Class of 2023, but feelings regarding the session were mixed. About half of the students found it to be helpful in guiding their decisions about joining opportunities in first year, while the other half found the session to be anxiety- and fear-inducing. To combat these feelings, a panel of fourth-year medical students was included in the MSPE introduction for the Class of 2024 to ease students’ anxiety and fears. However, only about half of the students found the panel useful. In response, the Global MedFIT Committee is currently devising a different method for introducing the MSPE to subsequent classes.

The evolution of MedFIT each year is also influenced by the pack mentors. For example, MedFIT 2017, 2018, and 2019 included anatomy, physiology, and pharmacology content from both the cardiovascular and neurology curricular blocks. Pack mentors for MedFIT 2020 recommended simplifying the curriculum to include only cardiovascular topics. The implementation of this change created a more cohesive program, allowing the pack mentors who planned MedFIT, the professors who taught lectures during MedFIT, and the matriculating students who went through MedFIT to focus on one topic instead of many.

In general, peer mentorship leaves students feeling more prepared, supported, and satisfied with their overall medical school experience when it is integrated appropriately and systematically from the start [11]. Table 2 discusses how pack mentors who are down-to-earth, approachable, and reliable generate an atmosphere focused on the idea of community rather than one of individualism. The longitudinal nature of this relationship encourages an open line of communication between mentors and mentees as they progress through their education and encounter new obstacles. Beyond this, COVID-19 continues to highlight the overarching principle that pack mentors are readily available to provide guidance, especially during times of uncertainty.

## Strengths, limitations, and future directions of MedFIT

One major strength of MedFIT is that a substantial portion of the program is organized by second-year pack mentors serving on sub-committees, which eases the workload on faculty who would have been responsible for organizing this large-scale event. Another strength of the MedFIT program includes its adaptability, proven by the efficient conversion of the program to a hybrid in-person/virtual format as a result of the COVID-19 pandemic. Additionally, the peer mentorship component of MedFIT fosters longitudinal mentor–mentee relationships and allows for continuous improvement based on the feedback received each year regarding student support.

One major limitation of this study is the lack of finalized longitudinal academic outcomes data. Although it is not feasible to finalize data sets for classes who have not yet or have only recently completed their program of study, incomplete data for certain classes does limit the ability to make conclusions about the impact of the MedFIT orientation program at large. Additionally, the fact that the MSBP program only included “at risk” students while MedFIT was required for the entire incoming class makes it difficult to directly compare outcomes. However, positive trends after the implementation of MedFIT can be seen when comparing them to average outcomes for MSBP students.

Other limitations of this program include logistics and finances. MedFIT requires organizing a dense schedule based on presenter/room/equipment availability. A pre-matriculation program of this caliber also requires a sizeable budget which should include funds for providing meals/snacks to students and staff as well as providing a stipend or some other form of compensation to more than twenty pack mentors.

Future directions of MedFIT include revisiting the longitudinal academic outcomes data once additional classes graduate to finalize incomplete information and implementing more quantitative assessments to determine the success of the program at large as well as the peer mentorship component. It would also be valuable to further assess other outcomes of the program including its impact on student confidence and comfort level with specialty selection and other important milestones in undergraduate medical education. Another future direction would be to further evaluate the impact of a hybrid MedFIT on this program's ability to prepare students for medical school when compared to the traditional, fully in-person format that was used prior to the pandemic.

## Conclusion

MedFIT puts an active twist on the passive delivery of a typical orientation for first-year students in an attempt to ease the transition into medical school. Since its implementation in 2017, MedFIT has grown into a much larger program, encompassing all departments at the medical school and even leading to the creation of a robust mentorship program between matriculating students and upperclassmen. The rigorous curriculum introduction, hands-on skills sessions, inviting faculty/staff, and passionate peer mentors are some of the many factors that have led to the high student satisfaction rate reported each year. Given the positive impact seen since its inception, MedFIT will continue as the premier pre-matriculation program at UNR Med for years to come.

## Supplementary Information

Below is the link to the electronic supplementary material.

Supplementary file1 (PDF 269 KB)

## Data Availability

Not applicable.
