# Supplementary material for: Developing a well-received pre-matriculation program: the evolution of MedFIT
Source: Discov Educ. 2022 Sep 15;1(1):12. doi: 10.1007/s44217-022-00012-z (PMC9476409; doi:10.1007/s44217-022-00012-z)
Supplement: Supplementary file 1 — Supplementary file1 (PDF 269 KB) [file 44217_2022_12_MOESM1_ESM.pdf]

University of Nevada, Reno
School of Medicine
Learning & Wellness Resource Center

Congratulations MedFIT Pack Mentors! This document provides the expectations and information regarding the responsibilities and tasks of MedFIT Pack Mentors. If you have any questions or concerns please let me know. This year, our MedFIT Pack Mentors will play a critical role in the development, implementation, and facilitation of different components of MedFIT. Because of the increased responsibilities, please understand that collaborative interprofessional teamwork is required of all MedFIT Pack Mentors.

Purpose Statement: As MedFIT Pack Mentors you are crucial to the success of MedFIT. You will have an opportunity to share with incoming MSIs what you have learned from your own experiences and make an impact on other students' success. You will have many opportunities to demonstrate how to be a successful medical student. Overall, you are a role model to the incoming class and will have an opportunity to teach, mentor, and directly work with the class of 2023.

Expectations & Responsibilities:

- As a MedFIT Pack Mentor, you are expected to attend all mandatory meetings. Most meetings are 1 hour in length and will not overlap with exam dates. These meetings are mandatory as we will provide crucial updates, plan major components of MedFIT, and discuss unexpected challenges and struggles. During these meetings we may also provide special training related to MedFIT. (Ex. training in art rounds or SP work)
- As a MedFIT Pack Mentor, you are expected to enter and submit all hours worked by the due date. Pack Mentors who submit their hours after the close of the pay period are not guaranteed payment for hours worked.
- As a MedFIT Pack Mentor, you may be asked to facilitate study groups, provide input for academic skills workshops, help participants learn good time management and study habits. Additionally, you may be asked to help students learn to understand academic expectations, manage stress, utilize campus resources, and balance academic and social obligation.
- As a MedFIT Pack Mentor, you will help assess participant's engagement and participation in MedFIT.
- As a MedFIT Pack Mentor, you will be provided with information so that you are well versed and knowledgeable about different resources available to our students. Pack Mentors are expected to share and relay this information even if it is a resource that you do not personally use. For example, during a note taking technique workshop, you may be asked to lead/facilitate a concept-mapping workshop. Even if you do not personally use this technique, our Pack Mentors are expected to teach how this tool may be effective. If you do not personally use this strategy you will have time to also share your personal tips with what has worked for you.
- Finally, MedFIT Pack Mentors are expected to have fun throughout the process. Please feel free to share your ingenuity, ideas, and input so that we can continue to make MedFIT an even greater experience!
- Your signature below indicates that you understand the expectations and time commitment required as a MedFIT Pack Mentor. If you are unable to meet the above-mentioned expectations, it is your responsibility to find a replacement by July 1, 2019.

____
Print Name

______________________________ ____________________
Signature Date
